# 2-(3-Chloro-1,2-dihydro­pyrazin-2-yl­idene)malononitrile

**DOI:** 10.1107/S1600536809006783

**Published:** 2009-02-28

**Authors:** Anita Stefańska, Tadeusz Ossowski, Artur Sikorski

**Affiliations:** a University of Gdańsk, Faculty of Chemistry, Sobieskiego 18/19, 80-952 Gdańsk, Poland

## Abstract

In the crystal structure of the title compound, C_7_H_3_ClN_4_, neighbouring mol­ecules are linked *via* pairs of N—H⋯N hydrogen bonds into inversion dimers, thereby forming an *R*_2_^2^(12) ring motif. With respective average deviations from planarity of 0.009 (1) and 0.006 (1) Å, the pyrazine skeleton and the malononitrile fragment are oriented at an angle of 6.0 (1)° with respect to each other. The mean planes of the pyrazine ring lie either parallel or are inclined at an angle of 68.5 (1)° in the crystal structure.

## Related literature

For applications of this class of compounds, see: Daniel *et al.* (1947 ▶); Dutcher (1947 ▶, 1958 ▶); Matter *et al.* (2005 ▶); Kaliszan *et al.* (1985 ▶); Lampen & Jones (1946 ▶); Petrusewicz *et al.* (1993 ▶, 1995 ▶); White (1940 ▶); White & Hill (1943 ▶). For related structures, see: Vishweshwar *et al.* (2000 ▶); Wardell *et al.* (2006 ▶). For the synthesis, see: Pilarski & Foks (1981 ▶, 1982 ▶). For the analysis of inter­molecular inter­actions, see: Spek (2009 ▶).
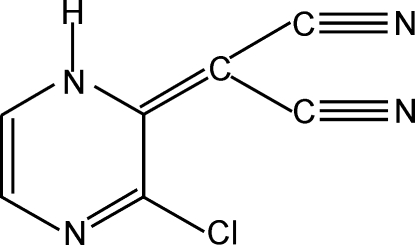


## Experimental

### 

#### Crystal data

C_7_H_3_ClN_4_
*M* *_r_* = 178.58
Monoclinic, 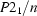

*a* = 5.7612 (2) Å
*b* = 8.1457 (2) Å
*c* = 16.2296 (5) Å
β = 94.116 (3)°
*V* = 759.67 (4) Å^3^
*Z* = 4
Mo *K*α radiation
μ = 0.44 mm^−1^
*T* = 295 K
0.40 × 0.10 × 0.08 mm

#### Data collection

Oxford Diffraction Ruby CCD diffractometer
Absorption correction: multi-scan (*CrysAlis RED*; Oxford Diffraction, 2008 ▶) *T*_min_ = 0.946, *T*_max_ = 0.967
6880 measured reflections
1335 independent reflections
1060 reflections with *I* > 2σ(*I*)
*R*_int_ = 0.026

#### Refinement

*R*[*F* ^2^ > 2σ(*F* ^2^)] = 0.030
*wR*(*F* ^2^) = 0.084
*S* = 1.10
1335 reflections
109 parameters
H-atom parameters constrained
Δρ_max_ = 0.15 e Å^−3^
Δρ_min_ = −0.19 e Å^−3^

### 

Data collection: *CrysAlis CCD* (Oxford Diffraction, 2008 ▶); cell refinement: *CrysAlis RED* (Oxford Diffraction, 2008 ▶); data reduction: *CrysAlis RED*; program(s) used to solve structure: *SHELXS97* (Sheldrick, 2008 ▶); program(s) used to refine structure: *SHELXL97* (Sheldrick, 2008 ▶); molecular graphics: *ORTEPII* (Johnson, 1976 ▶); software used to prepare material for publication: *SHELXL97* and *PLATON* (Spek, 2009 ▶).

## Supplementary Material

Crystal structure: contains datablocks I, global. DOI: 10.1107/S1600536809006783/xu2486sup1.cif

Structure factors: contains datablocks I. DOI: 10.1107/S1600536809006783/xu2486Isup2.hkl

Additional supplementary materials:  crystallographic information; 3D view; checkCIF report

## Figures and Tables

**Table 1:** Hydrogen-bond geometry (Å, °)

*D*—H⋯*A*	*D*—H	H⋯*A*	*D*⋯*A*	*D*—H⋯*A*
N1—H1⋯N9^i^	0.86	2.10	2.896 (2)	154

Symmetry code: (i) 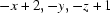.

**Fig. 1.**
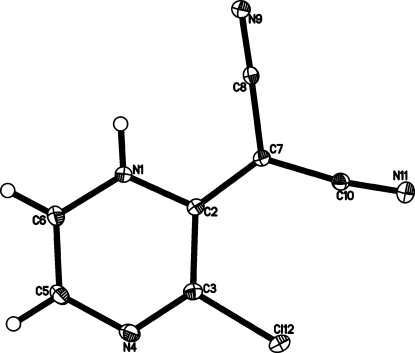
The molecular structure of the title compound showing the atom-labeling scheme. Displacement ellipsoids are drawn at the 25% probability level and H atoms are shown as small spheres of arbitrary radius.

**Fig. 2.**
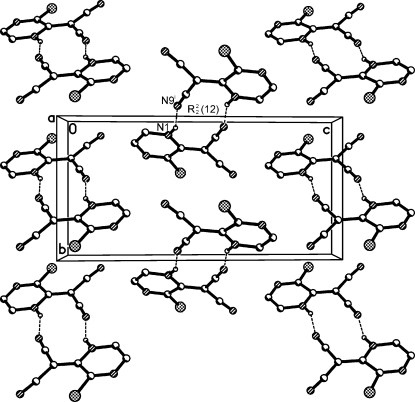
The arrangement of the molecules in the crystal structure viewed approximately along a axis. The N—H···N interactions are represented by dashed lines. H atoms not involved in the interactions have been omitted. [Symmetry codes: (i) 2 - x, - y, 1 - z.]

## supplementary crystallographic information

### Comment

The pyrazine ring is found in many physiologically active compounds, including natural products such as folic acid (Lampen & Jones, 1946), aspergillic acid (Dutcher, 1947), pterins (Daniel *et al.*, 1947; Matter *et al.*, 2005). Important group of natural compounds are derivatives which posses antibiotic activities, for examples aspegillic acid isolated from *Aspergillus flavus* (Dutcher, 1958; White, 1940; White & Hill, 1943). Some of pyrazine-acetonitrile compounds also posses biological activities. Some of them show anti-inflammatory (Petrusewicz *et al.*, 1995) and analgesic activities (Kaliszan *et al.*, 1985; Petrusewicz *et al.*, 1993). We decided to synthesis some of this derivatives. 2-(3-Chloropyrazin-2(1H)-ylidene)malononitrile belongs to pyrazine-acetonitrile derivatives. We report here crystal structure of the title compound, 2-(3-chloropyrazin-2(1H)-ylidene)malononitrile.

In the molecule of the title compound (Fig. 1) the bond lengths and angles characterizing the geometry of the pyrazines skeleton are typical for this group compounds (Vishweshwar *et al.*, 2000; Wardell *et al.*, 2006). With respective average deviations from planarity of 0.009 (1) and 0.006 (1) Å, the pyrazine skeleton and malononitrile fragment are oriented at an angle 6.0 (1)° to each other. The mean planes of the pyrazine skeleton lie either parallel or are inclined at an angle of 68.5 (1)° in the lattice. One of the nitrile fragment (delineated by C7, C8 and N9 atoms) is nearly in the plane of the heterocyclic ring (the angle between the mean planes of the pyrazine skeleton and nitrile fragment is equal 178.4 (2)°) while the other (involving C7, C10 and N11 atoms) is out of plane the pyrazine skeleton (the angle between the mean planes of the pyrazine skeleton and nitrile fragment is equal 172.8 (2)°).

In the crystal structure, neighbouring molecules are linked through N–H···N hydrogen bond forming R_2_^2^(12) ring motif (Table 1 and Fig. 2). The interactions demonstrated were found by *PLATON* (Spek, 2009).

### Experimental

2-[3-Chloropyrazin-2(1H)-ylidene)malononitrile was obtained by the aromatic nucleophilic substitution of chlorine in 2,3-dichloropyrazine with malononitrile (Pilarski & Foks, 1981 and 1982). A mixture of 2,3-dichloropyrazine, malononitrile and potassium carbonate was dissolved in DMSO. The mixture was stirred for 4 h in 333 K to give an orange solution. After cooling the reaction mixture to room temperature, water was added. Then mixture was acidified with hydrochloric acid. Single crystals suitable for X-ray analysis were grown in methanol solution [m.p. = 436 K].

### Refinement

All H atoms were positioned geometrically and refined using a riding model, with C–H = 0.93 Å and *U*_iso_(H) = 1.2*U*_eq_(C) and N–H = 0.86 Å and *U*_iso_(H) = 1.2*U*_eq_(N).

### Crystal data

**Table:**

C_7_H_3_ClN_4_	*F*(000) = 360
*M**_r_* = 178.58	*D*_x_ = 1.561 Mg m^−^^3^
Monoclinic, *P*2_1_/*n*	Mo *K*α radiation, λ = 0.71073 Å
Hall symbol: -P 2yn	Cell parameters from 1335 reflections
*a* = 5.7612 (2) Å	θ = 3.0–25.0°
*b* = 8.1457 (2) Å	µ = 0.44 mm^−^^1^
*c* = 16.2296 (5) Å	*T* = 295 K
β = 94.116 (3)°	Needle, orange
*V* = 759.67 (4) Å^3^	0.40 × 0.10 × 0.08 mm
*Z* = 4	

### Data collection

**Table:**

Oxford Diffraction Ruby CCD diffractometer	1335 independent reflections
Radiation source: Enhance (Mo) X-ray Source	1060 reflections with *I* > 2σ(*I*)
graphite	*R*_int_ = 0.026
Detector resolution: 10.4002 pixels mm^-1^	θ_max_ = 25.0°, θ_min_ = 3.6°
ω scans	*h* = −6→6
Absorption correction: multi-scan (*CrysAlis RED*; Oxford Diffraction, 2008)	*k* = −9→9
*T*_min_ = 0.946, *T*_max_ = 0.967	*l* = −18→19
6880 measured reflections	

### Refinement

**Table:**

Refinement on *F*^2^	Secondary atom site location: difference Fourier map
Least-squares matrix: full	Hydrogen site location: inferred from neighbouring sites
*R*[*F*^2^ > 2σ(*F*^2^)] = 0.030	H-atom parameters constrained
*wR*(*F*^2^) = 0.084	*w* = 1/[σ^2^(*F*_o_^2^) + (0.0481*P*)^2^ + 0.0305*P*] where *P* = (*F*_o_^2^ + 2*F*_c_^2^)/3
*S* = 1.10	(Δ/σ)_max_ < 0.001
1335 reflections	Δρ_max_ = 0.14 e Å^−^^3^
109 parameters	Δρ_min_ = −0.19 e Å^−^^3^
0 restraints	Extinction correction: *SHELXL97* (Sheldrick, 2008)
Primary atom site location: structure-invariant direct methods	

### Special details

**Table:**

Geometry. All esds (except the esd in the dihedral angle between two l.s. planes) are estimated using the full covariance matrix. The cell esds are taken into account individually in the estimation of esds in distances, angles and torsion angles; correlations between esds in cell parameters are only used when they are defined by crystal symmetry. An approximate (isotropic) treatment of cell esds is used for estimating esds involving l.s. planes.
Refinement. Refinement of *F*^2^ against ALL reflections. The weighted *R*-factor *wR* and goodness of fit *S* are based on *F*^2^, conventional *R*-factors *R* are based on *F*, with *F* set to zero for negative *F*^2^. The threshold expression of *F*^2^ > σ(*F*^2^) is used only for calculating *R*-factors(gt) *etc*. and is not relevant to the choice of reflections for refinement. *R*-factors based on *F*^2^ are statistically about twice as large as those based on *F*, and *R*- factors based on ALL data will be even larger.

### Fractional atomic coordinates and isotropic or equivalent isotropic displacement parameters (Å^2^)

**Table:**

	*x*	*y*	*z*	*U*_iso_*/*U*_eq_	
N1	0.6636 (3)	0.11965 (16)	0.38343 (8)	0.0365 (4)	
H1	0.7797	0.0722	0.4098	0.044*	
C2	0.5179 (3)	0.20873 (19)	0.42756 (10)	0.0313 (4)	
C3	0.3266 (3)	0.2760 (2)	0.37691 (10)	0.0373 (4)	
N4	0.2971 (3)	0.25960 (19)	0.29792 (10)	0.0523 (5)	
C5	0.4568 (4)	0.1719 (3)	0.25856 (12)	0.0611 (6)	
H5	0.4395	0.1613	0.2014	0.073*	
C6	0.6386 (4)	0.1004 (2)	0.30035 (11)	0.0508 (5)	
H6	0.7452	0.0389	0.2730	0.061*	
C7	0.5634 (3)	0.22285 (19)	0.51338 (9)	0.0327 (4)	
C8	0.7584 (3)	0.13941 (19)	0.55144 (10)	0.0343 (4)	
N9	0.9152 (3)	0.0691 (2)	0.58089 (9)	0.0467 (4)	
C10	0.4414 (3)	0.3217 (2)	0.56834 (11)	0.0383 (4)	
N11	0.3624 (3)	0.3981 (2)	0.61843 (11)	0.0568 (5)	
Cl12	0.11485 (8)	0.38509 (6)	0.42290 (3)	0.0490 (2)	

### Atomic displacement parameters (Å^2^)

**Table:**

	*U*^11^	*U*^22^	*U*^33^	*U*^12^	*U*^13^	*U*^23^
N1	0.0352 (9)	0.0416 (8)	0.0323 (8)	0.0067 (7)	0.0001 (6)	0.0014 (6)
C2	0.0292 (9)	0.0286 (8)	0.0364 (9)	−0.0016 (7)	0.0034 (7)	0.0010 (7)
C3	0.0358 (10)	0.0344 (9)	0.0413 (10)	0.0015 (8)	−0.0009 (8)	0.0016 (7)
N4	0.0591 (12)	0.0544 (10)	0.0416 (9)	0.0133 (9)	−0.0099 (8)	−0.0002 (7)
C5	0.0793 (17)	0.0700 (14)	0.0324 (10)	0.0224 (13)	−0.0072 (11)	−0.0037 (9)
C6	0.0608 (14)	0.0565 (11)	0.0351 (10)	0.0137 (10)	0.0041 (9)	−0.0041 (8)
C7	0.0303 (10)	0.0330 (8)	0.0348 (9)	0.0012 (7)	0.0017 (7)	0.0013 (7)
C8	0.0379 (11)	0.0348 (9)	0.0302 (9)	−0.0012 (8)	0.0037 (8)	−0.0034 (7)
N9	0.0460 (11)	0.0536 (9)	0.0397 (9)	0.0108 (8)	−0.0029 (8)	−0.0034 (7)
C10	0.0341 (11)	0.0440 (10)	0.0366 (10)	0.0007 (8)	0.0016 (8)	0.0025 (8)
N11	0.0539 (11)	0.0717 (11)	0.0456 (10)	0.0135 (9)	0.0088 (8)	−0.0075 (8)
Cl12	0.0368 (3)	0.0530 (3)	0.0568 (3)	0.0114 (2)	0.0017 (2)	0.0011 (2)

### Geometric parameters (Å, °)

**Table:**

N1—C2	1.353 (2)	C5—C6	1.339 (3)
N1—C6	1.355 (2)	C5—H5	0.9300
N1—H1	0.8600	C6—H6	0.9300
C2—C7	1.403 (2)	C7—C8	1.416 (2)
C2—C3	1.436 (2)	C7—C10	1.424 (2)
C3—N4	1.288 (2)	C8—N9	1.145 (2)
C3—Cl12	1.7219 (18)	C10—N11	1.144 (2)
N4—C5	1.360 (3)		
			
C2—N1—C6	124.28 (15)	C6—C5—H5	119.3
C2—N1—H1	117.9	N4—C5—H5	119.3
C6—N1—H1	117.9	C5—C6—N1	118.50 (18)
N1—C2—C7	119.36 (15)	C5—C6—H6	120.8
N1—C2—C3	112.43 (15)	N1—C6—H6	120.8
C7—C2—C3	128.20 (16)	C2—C7—C8	118.66 (14)
N4—C3—C2	124.83 (17)	C2—C7—C10	127.03 (15)
N4—C3—Cl12	116.00 (14)	C8—C7—C10	114.20 (14)
C2—C3—Cl12	119.16 (13)	N9—C8—C7	178.40 (18)
C3—N4—C5	118.48 (16)	N11—C10—C7	172.83 (19)
C6—C5—N4	121.43 (18)		
			
C6—N1—C2—C7	−178.76 (16)	C3—N4—C5—C6	1.5 (3)
C6—N1—C2—C3	2.4 (2)	N4—C5—C6—N1	−1.2 (3)
N1—C2—C3—N4	−2.1 (3)	C2—N1—C6—C5	−0.9 (3)
C7—C2—C3—N4	179.14 (17)	N1—C2—C7—C8	−1.6 (2)
N1—C2—C3—Cl12	176.92 (11)	C3—C2—C7—C8	177.09 (16)
C7—C2—C3—Cl12	−1.8 (3)	N1—C2—C7—C10	174.39 (16)
C2—C3—N4—C5	0.3 (3)	C3—C2—C7—C10	−6.9 (3)
Cl12—C3—N4—C5	−178.77 (15)		

### Hydrogen-bond geometry (Å, °)

**Table:**

*D*—H···*A*	*D*—H	H···*A*	*D*···*A*	*D*—H···*A*
N1—H1···N9^i^	0.86	2.10	2.896 (2)	154

Symmetry codes: (i) −*x*+2, −*y*, −*z*+1.
